# HuangLian-4 alleviates myocardial ischemia-reperfusion injury by activating the pro-survival STAT3 signaling pathway

**DOI:** 10.3389/fphar.2025.1683575

**Published:** 2025-11-05

**Authors:** Chelimuge Gong, Dalintai Wang, Qingshan Zhang

**Affiliations:** ^1^ College of Clinical Medicine, Inner Mongolia Minzu University, Tongliao, China; ^2^ Inner Mongolia Minzu University, Tongliao, China

**Keywords:** myocardial ischemia-reperfusion injury, HuangLian-4, stat3, H9c2 cardiomyocytes, network pharmacology

## Abstract

Myocardial ischemia-reperfusion injury (MIRI) remains a major clinical challenge following revascularization for acute myocardial infarction. The multi-component, multi-target nature of traditional herbal formulas like HuangLian-4 (HL4) offers a promising therapeutic paradigm for this complex disease, yet its underlying molecular mechanism is poorly understood. This study aimed to systematically elucidate the cardioprotective mechanism of HL4 by integrating serum pharmacochemistry, network pharmacology, and systems biology with experimental validation. An *in vitro* MIRI model was established using a hypoxia/reoxygenation (H/R) protocol in H9c2 cardiomyocytes, and the effects of HL4 were assessed by ELISA, qRT-PCR, Western blot, and molecular docking. Our results demonstrate that HL4 treatment significantly attenuated H/R-induced cardiomyocyte injury and inflammation, reducing the release of CK-MB, LDH, IL-6, and TNF-α. Mechanistically, our primary finding revealed that HL4 markedly rescued the H/R-induced downregulation of the pro-survival factor STAT3 at both the mRNA and protein levels. This activation of STAT3 was accompanied by a significant increase in the anti-apoptotic protein Bcl-2 and a decrease in pro-apoptotic Bax, alongside the suppression of the maladaptive factor HIF-1α. These experimental findings were powerfully corroborated by systems-level bioinformatic analysis, which independently identified the STAT family as key upstream regulators of a MIRI-critical gene network. Furthermore, molecular docking confirmed a strong binding affinity between key active compounds of HL4 and the STAT3 protein. In conclusion, this study demonstrates that HL4 alleviates MIRI by activating the STAT3 signaling pathway, which in turn orchestrates a downstream anti-apoptotic program. Our findings provide a robust molecular rationale for the clinical use of HL4 and establish STAT3 modulation as a promising therapeutic strategy for MIRI.

## 1 Introduction

Acute myocardial infarction (AMI), a critical cardiac emergency caused by the abrupt occlusion of coronary arteries, stands as a primary driver of global cardiovascular mortality, accounting for approximately half of the 18 million cardiovascular-related deaths each year (Saito et al., 2023; Algoet et al., 2023). The cornerstone of modern AMI treatment is rapid revascularization, most effectively achieved through percutaneous coronary intervention (PCI). While this procedure is essential for salvaging ischemic tissue, the very act of restoring blood flow paradoxically initiates a secondary pathological process known as myocardial ischemia-reperfusion injury (MIRI) (Zhang et al., 2024). This complex injury cascade, manifesting as reperfusion arrhythmias, myocardial stunning, and microvascular obstruction, can negate the benefits of revascularization, contributing significantly to final infarct size and long-term adverse cardiac remodeling. Consequently, mitigating MIRI remains one of the most significant unmet challenges in contemporary cardiology.

The pathophysiology of MIRI is notoriously complex, orchestrated by a web of interconnected events including massive bursts of reactive oxygen species, intracellular calcium overload, an intense inflammatory response, and, critically, cardiomyocyte apoptosis, which is a key driver of irreversible cell loss and cardiac dysfunction (Algoet et al., 2023). This multifactorial nature explains the disappointing results of numerous clinical trials targeting single molecular pathways. Therapies focusing on individual mechanisms—such as antioxidants, anti-inflammatory agents, or apoptosis inhibitors—have largely failed to translate into meaningful clinical benefits, often due to insufficient efficacy or poor specificity (Vallejo et al., 2022). This persistent translational failure underscores a fundamental mismatch between single-target drugs and a multi-faceted disease, highlighting a critical research gap: the need for novel therapeutic agents that can simultaneously engage multiple key nodes within the MIRI pathological network (Frangogiannis, 2014; Chen et al., 2014).

Traditional herbal medicine formulas, with their inherent multi-component, multi-target nature, offer a compelling therapeutic paradigm precisely suited to tackling such complex diseases. Among these, the traditional Mongolian medicine prescription HuangLian-4 (HL4) has been historically used for cardiovascular-related ailments (Ding et al., 2024). Modern, albeit limited, research has provided preliminary support for its efficacy; previous studies have shown that HL4 can attenuate the inflammatory markers TNF-α and IL-6 in a rat model of MIRI, and that its principal herb contains compounds with direct anti-apoptotic effects on cardiomyocytes (Liu et al., 2016; Xiao, 2018). However, these initial observations are largely descriptive and fragmented. The specific bioavailable compounds responsible for its therapeutic effect, the key molecular targets they engage, and the overarching signaling pathway that governs its potent anti-apoptotic action remain a critical “black box,” preventing a deep understanding of its pharmacological basis.

In this study, we employ an integrative research strategy that combines serum pharmacochemistry to identify bioavailable constituents of the HL4 formula, network pharmacology to predict their putative targets and pathways, and systems biology—specifically Weighted Gene Co-expression Network Analysis (WGCNA)—to identify disease-relevant gene modules and regulatory hubs. This computational framework is further substantiated through *in vitro* validation using a hypoxia/reoxygenation-induced cardiomyocyte injury model. By bridging traditional medicinal knowledge with multi-dimensional scientific methodologies, this approach provides a robust platform for elucidating the mechanistic basis of multi-component herbal formulations and offers a valuable reference for the development of multi-targeted therapeutic strategies for complex cardiovascular disorders such as myocardial ischemia-reperfusion injury.

## 2 Materials and methods

### 2.1 Reagents and antibodies

Chemicals and reagents included: pepsin (P8390; Solarbio, Beijing, China), fetal bovine serum (FBS) (2414839RP; Gibco, Beijing, China), DMEM high glucose medium (8122554; Gibco, Beijing, China), penicillin-streptomycin solution (referred to as cyanomycin double antibody) (10378016; Gibco, Beijing, China), *mycoplasma* prophylactic reagent (FM501; Quanshi Gold Biotechnology, Beijing, China), 0.25% trypsin-EDTA (25200-056; Gibco, Beijing, China), DMSO (D8371; Solarbio, Beijing, China), PBS (P1020; Solarbio, Beijing, China), and TRIzol Reagent (CW0580S; Kangwei Century, Jiangsu, China). The CCK-8 kit (CA1210-500) was from Solarbio. A mixed gas (94% N2, 5% CO2, 1% O2) was supplied by the Mongolian Medicine Functional Research and Development Laboratory.

ELISA kits for Lactate dehydrogenase (LDH) (ZY3480-A), CK-MB (ZY3500-A2), IL-6 (ZY3066-A), and TNF-α (ZY3056-A) were purchased from Zeyuwu Technology Co., Ltd. (Jiangsu, China).

Primary antibodies used were: anti-Bax (50599-2-Ig; Proteintech, Wuhan, China), anti-Bcl-2 (26593-1-AP; Proteintech), anti-HIF-1α (20960-1-AP; Proteintech), and anti-STAT3 (E20-53502; ENOGENE, Shanghai, China). Secondary antibodies included goat anti-mouse IgG (D110087-0100; BBI, Shanghai, China) and goat anti-rabbit IgG (D110058-0001; BBI, Shanghai, China). The anti-GAPDH antibody (D190090-0100; BBI) was used as a loading control.

Molecular biology kits included a qPCR kit (AQ132) and a cDNA synthesis kit (AT341) from Quanshi Gold Biotechnology (Beijing, China). A whole protein extraction kit (BC3710) was from Solarbio, and a BCA protein detection kit (DQ111) was from TransGen Biotech (Beijing, China).

### 2.2 HL4 decoction and serum pharmacochemistry

The HuangLian-4 (HL4) decoction (Batch No. 20191201) was prepared and supplied by the Affiliated Hospital of Inner Mongolia University for Nationalities (Inner Mongolia, China).

Twelve male Balb/c mice were obtained from Shenyang Changsheng Laboratory Animal Co., Ltd. All experimental procedures were approved by the Institutional Animal Care and Use Committee of Inner Mongolia Minzu University (License No.: SCXK-(Liao)-2020-0001) and were carried out in compliance with animal welfare ethical review guidelines. The mice were randomly allocated to a blank control group (n = 6) or an HL4 decoction group (n = 6). The HL4 group received a daily oral gavage of the decoction at a dose equivalent to 3 g/day for a 70 kg human. The control group received an equivalent volume of distilled water. After 7 consecutive days of administration, with a 12-h fast after the day 6 dose, mice were gavaged a final time on day 7. Blood was collected from the orbital venous plexus, allowed to stand overnight at 4 °C, and then centrifuged at 3,000 rpm for 15 min to isolate serum. Serum from each group was pooled, heat-inactivated at 56 °C for 30 min, and sterilized by filtration through a 0.22 µm membrane. Aliquots were stored at −80 °C.

For analysis, pooled serum was thawed. To a 500 µL aliquot, 1.5 mL of methanol-acetonitrile (1:1, v/v) was added to precipitate proteins. The mixture was vortexed for 3 min and centrifuged at 15,000 rpm for 10 min at 4 °C. The supernatant was dried under nitrogen, reconstituted in 200 µL of methanol, sonicated for 3 min (250 W, 40 kHz), vortexed for 1 min, and centrifuged again (15,000 rpm, 10 min, 4 °C). The final supernatant was collected for analysis.

### 2.3 UPLC-QTOF-MS analysis

Samples were analyzed on a Thermo Orbitrap Exploris 120 mass spectrometer with an electrospray ionization (ESI) source operating in both positive and negative ion modes. Key parameters were: spray voltage, +3.50 kV (positive) and −2.50 kV (negative); sheath gas pressure, 30 arb; auxiliary gas pressure, 10 arb; capillary temperature, 325 °C. Full scan MS1 spectra were acquired from m/z 100 to 1000 at a resolution of 60,000. MS2 fragmentation was performed using high-energy collisional dissociation (HCD) with a normalized collision energy of 30% and a resolution of 15,000. The top four most intense ions were selected for fragmentation, with dynamic exclusion enabled. Metabolite identification was based on exact mass (error ≤30 ppm) and MS/MS fragmentation patterns matched against public (Human Metabolome Database, LipidMaps, mzCloud) and in-house databases.

### 2.4 Network pharmacology analysis

Potential targets for HL4 compounds were predicted using Swiss Target Prediction, Similarity Ensemble Approach (SEA), SuperPred, and PubChem. Disease targets for “myocardial ischemia-reperfusion injury” were retrieved from GeneCards and OMIM. Intersecting targets were identified using R (v. 4.2.1). A drug-component-target network was visualized using Cytoscape (v. 3.10.2). Protein-protein interaction (PPI) analysis was performed using the STRING database (v. 11.5) with a high confidence score (>0.9) for *Homo sapiens*, and hub genes were identified with the CytoHubba plugin. GO and KEGG pathway enrichment analyses were performed in R.

### 2.5 Systems biology analysis (WGCNA) and upstream regulator prediction

To provide an independent, systems-level validation of our network pharmacology findings, a Weighted Gene Co-expression Network Analysis (WGCNA) was performed on the MIRI-related gene expression dataset GSE1089_40, obtained from the Gene Expression Omnibus (GEO) database. The WGCNA R package was used to construct a gene co-expression network. An appropriate soft-thresholding power of β = 8 was selected to ensure a scale-free network topology, as determined by the scale independence and mean connectivity analysis (Supplementary Figures S1A, S1B). Subsequently, key modules significantly associated with the MIRI phenotype were identified, and core genes were screened. Furthermore, to explore the upstream regulatory mechanisms of the MIRI-critical module, transcription factor (TF) enrichment analysis was conducted using the msigdbr and clusterProfiler R packages based on the GTRD and TFT_LEGACY databases.

### 2.6 Molecular docking simulation

To predict the binding affinity and interaction modes between key active compounds of HL4 and core target proteins, molecular docking simulations were performed. The three-dimensional (3D) structures of four representative compounds (Dapsone, swertiamarin, Thiocticacid, Gentiopicrin) were obtained from the PubChem database. The 3D crystal structures of the receptor proteins—BAX (PDB: 4S0O), BCL2 (PDB: 2W3L), STAT3 (PDB: 6NJS), and HIF-1α (PDB: 4H6J)—were downloaded from the RCSB Protein Data Bank.

Prior to docking, all receptor proteins were prepared by removing water molecules and original ligands and adding polar hydrogen atoms using PyMOL software. The docking procedure was then carried out using the CB-dock2 online server, which employs AutoDock Vina for calculation. The binding affinity for each compound-protein pair was evaluated based on the Vina score (kcal/mol), where a lower score indicates a more stable interaction. The optimal docking conformation with the lowest binding energy was selected for visualizing the 2D and 3D protein-ligand interactions.

### 2.7 Cell culture and hypoxia/reoxygenation (H/R) model

H9c2 rat cardiomyoblasts (Beina C&L Biotechnology, China) were cultured in high-glucose DMEM (4.5 g/L glucose) supplemented with 10% FBS, 50 U/mL penicillin, and 50 μg/mL streptomycin in a standard humidified incubator at 37 °C with an atmosphere of 95% air and 5% CO_2_. For the hypoxia phase, the standard medium was replaced with serum-free high-glucose DMEM. To establish the *in vitro* model of myocardial ischemia/reperfusion injury, cells were grouped as follows: a control group, a model group (H/R), and an HL4 treatment group. The HL4 group was pre-treated with the optimal concentration of HL4 (150 μg/mL) for 24 h. Subsequently, both the model and HL4 treatment groups were subjected to hypoxia by incubation in a three-gas incubator (1% O_2_, 5% CO_2_, 94% N_2_) for 3 h. This was followed by 3 h of reoxygenation, during which the cells were returned to the standard normoxic incubator. The control group remained in the normoxic incubator for the entire duration of the experiment.

### 2.8 Cell viability assay for optimal concentration screening

To determine the optimal therapeutic concentration of HL4, a cell viability assay was performed. Cells were seeded in 96-well plates at 5,000 cells/well and allowed to adhere for 24 h. The medium was then replaced with fresh medium containing a gradient of HL4 extract concentrations (0, 50, 100, 150, 200, 250, 300, 350, and 400 μg/mL) and incubated for 24 h. Following treatment, the medium was replaced with 90 µL of fresh medium and 10 µL of CCK-8 reagent. After a 2-h incubation in the dark, absorbance was measured at 450 nm. The concentration that induced the highest cell proliferation without showing cytotoxicity was selected for subsequent experiments.

### 2.9 Enzyme-linked immunosorbent assay (ELISA)

Cell culture supernatant was centrifuged at 3,000 rpm for 20 min at room temperature. The levels of CK-MB, LDH, IL-6, and TNF-α in the supernatant were measured using commercial ELISA kits according to the manufacturer’s instructions (Zeyuwu Technology Co., Ltd.).

### 2.10 Quantitative real-time PCR (qRT-PCR)

Total RNA was extracted from cells using TRIzol reagent. 1 μg of total RNA was reverse-transcribed into cDNA using a commercial kit. qPCR was performed using specific primers (Table 1) with GAPDH as the internal control. The thermal cycling protocol was: 95 °C for 30 s, followed by 40 cycles of 95 °C for 5 s and 60 °C for 40 s. Relative gene expression was calculated using the 2^−^ΔΔCt method. Experiments were performed in triplicate.

**TABLE 1:** Primer sequences used for qRT-PCR.

Gene	Forward primer (5′-3′)	Reverse primer (5′-3′)
Bax	GAC​GCA​TCC​ACC​AAG​AAG​CTG​AG	GCT​GCC​ACA​CGG​AAG​AAG​ACC
Bcl-2	TGG​AGA​GCG​TCA​ACA​GGG​AGA​TG	GGT​GTG​CAG​ATG​CCG​GTT​CAG
HIF-1α	CTC​ACA​GTC​GGA​CAA​CCT​CA	TGC​TGC​AGT​AAC​GTT​CCA​ATT​C
STAT3	AGC​AGC​CAA​ACT​CCC​AGA​TC	ATC​AGC​TCA​CAG​AAA​GGG​GC
GAPDH	AGG​TGA​CCG​CAT​CTT​CTT​GT	TAC​GGC​CAA​ATC​CGT​TCA​CA

### 2.11 Western blot analysis

Total protein was extracted using a commercial kit and quantified with a BCA assay. 20 μg of protein per lane was separated on a 10% SDS-PAGE gel and transferred to a PVDF membrane (Millipore, IPVH00010). The membrane was blocked with 5% non-fat milk in TBS-T for 1 h at room temperature. Membranes were incubated overnight at 4 °C with primary antibodies against Bax (1:1000), Bcl-2 (1:1000), HIF-1α (1:500), STAT3 (1:500), and GAPDH (1:1000). After washing, membranes were incubated with fluorescent secondary antibodies for 1 h at room temperature. Chemiluminescent signals were detected using the ChemiDoc XRS imaging system (Bio-Rad) and band intensities were quantified using ImageJ software. Experiments were performed in triplicate.

### 2.12 Statistical analysis

All statistical analyses were performed using GraphPad Prism (v. 9.5.1). Data are presented as mean ± standard error of the mean (SEM). Comparisons between two groups were made using an unpaired Student’s t-test. Differences among multiple groups were analyzed using one-way analysis of variance (ANOVA). A p-value of <0.05 was considered statistically significant.

## 3 Results

### 3.1 Identification of blood-absorbed components of HL4 by UPLC-QTOF-MS

**FIGURE 1:**
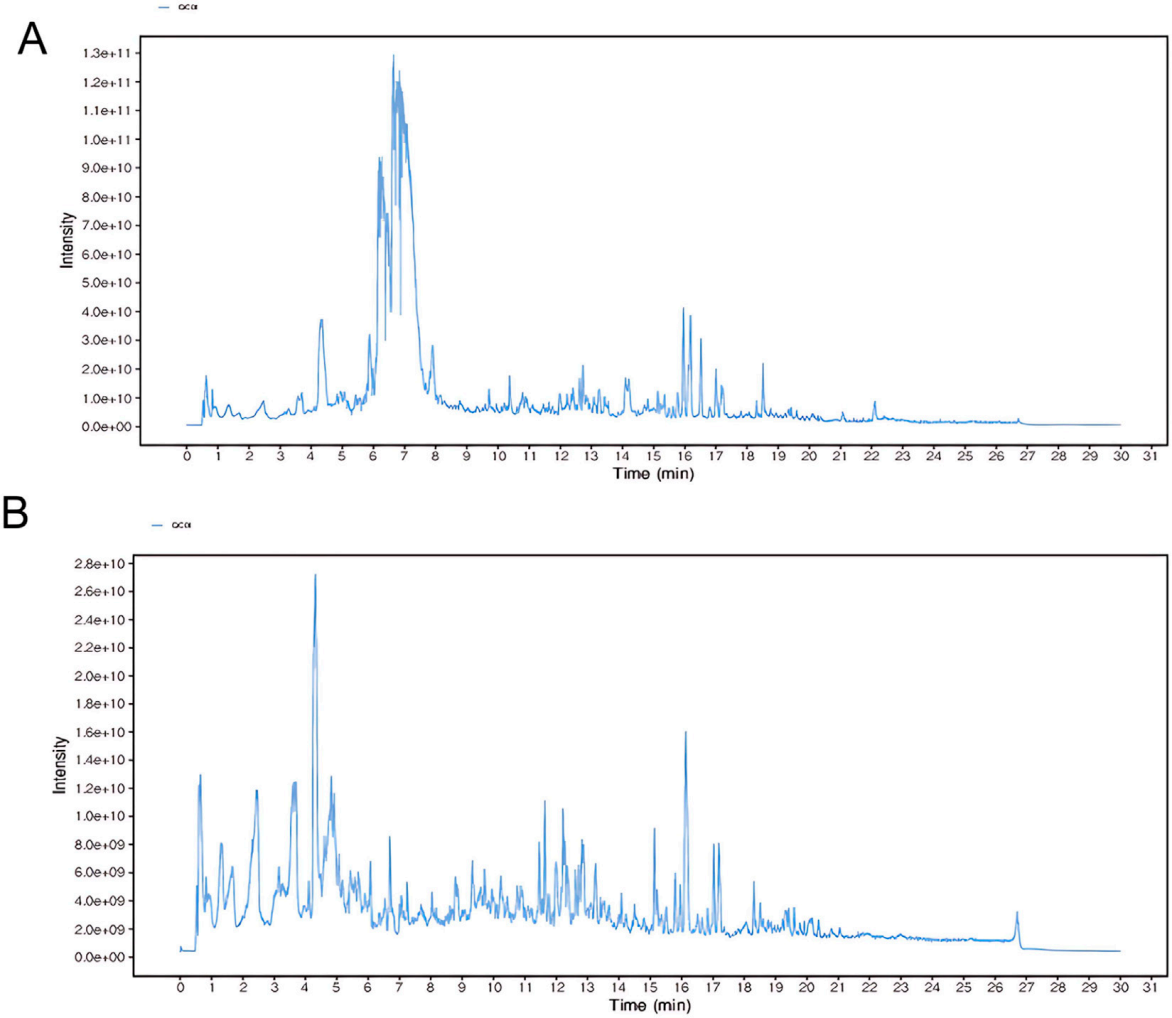
Representative UPLC-QTOF-MS total ion chromatograms (TICs) of HL4-medicated mouse serum. The chromatograms show the complex profile of blood-absorbed components from HL4. **(A)** TIC obtained in positive electrospray ionization (ESI+) mode, from which 24 components were identified. **(B)** TIC obtained in negative electrospray ionization (ESI−) mode, from which 16 components were identified.

To identify the potentially bioactive constituents of HL4 that enter the bloodstream following oral administration, serum from HL4-treated mice was analyzed by UPLC-QTOF-MS. The representative total ion chromatograms (TICs) acquired in both positive and negative electrospray ionization (ESI) modes are presented in Figure 1. The chromatograms reveal a highly complex chemical profile, indicative of the multi-component nature of the absorbed HL4 constituents and their metabolites. In the positive ion mode (Figure 1A), the TIC was characterized by a dense cluster of high-intensity peaks eluting predominantly between 5.0 and 8.0 min, which contained the majority of the total ion signal. Numerous other lower-intensity peaks were distributed across the entire chromatographic run, highlighting the chemical diversity of the compounds ionized in this mode. Through meticulous data processing and database matching, a total of 24 distinct components were successfully identified from the positive ion mode data. In contrast, the TIC from the negative ion mode (Figure 1B) displayed a different chromatographic profile. While the overall signal intensity was lower than in the positive mode, a broad distribution of well-separated peaks was observed from approximately 1.0–20.0 min. This mode was effective in capturing a distinct set of compounds, likely those with acidic functional groups that are more readily ionized under negative ESI conditions. From this analysis, 16 additional components were identified.

Collectively, the comprehensive, dual-mode analysis led to the successful identification of 40 unique blood-absorbed components from the HL4-medicated serum. These compounds, which included various classes such as alkaloids, terpenoids, and phenylpropanoids, were considered the potential material basis for HL4’s therapeutic effects. These identified constituents were subsequently used for network pharmacology analysis to predict their molecular targets and elucidate the underlying mechanisms of action against myocardial ischemia-reperfusion injury.

### 3.2 Network pharmacology analysis identifies key targets and pathways of HL4 in MIRI

**FIGURE 2:**
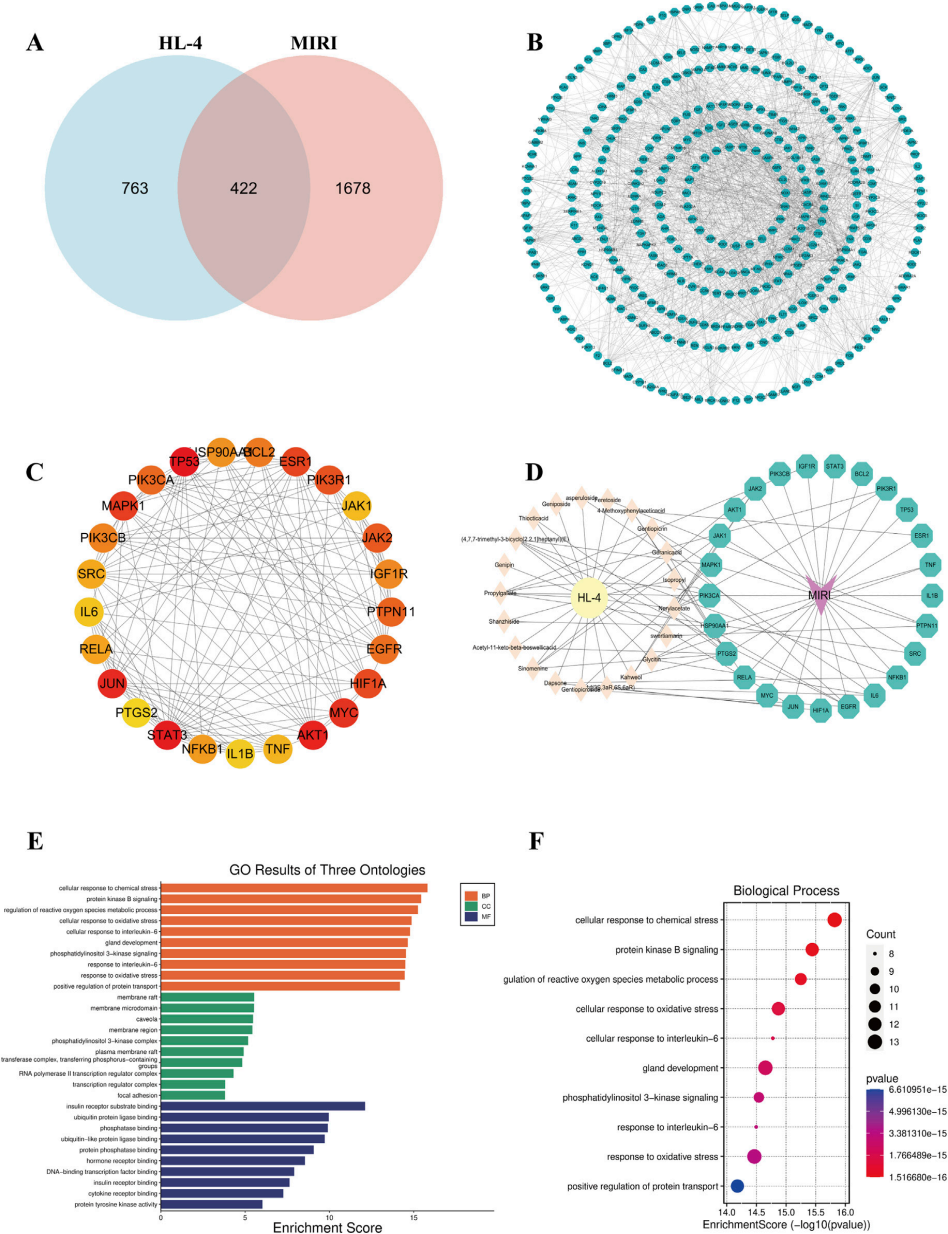
Network pharmacology analysis of HL4 for the treatment of MIRI. **(A)** Venn diagram showing the intersection of 763 HL4-related targets and 1678 MIRI-related targets, yielding 422 common targets. **(B)** The comprehensive PPI network constructed from the 422 common targets. **(C)** A subnetwork highlighting the top-ranking hub genes, with node color indicating degree centrality (red = high, yellow = low). **(D)** The “Drug-Component-Target-Disease” network illustrating the interactions between active components in HL4 and core MIRI targets. **(E)** Bar plot of the top enriched GO terms, categorized by BP, CC, and MF. **(F)** Bubble plot of the top 10 most significantly enriched GO Biological Process terms. The size of the bubbles represents the gene count, and the color represents the P-value.

To systematically uncover the potential molecular mechanisms of HL4 in treating MIRI, we conducted a comprehensive network pharmacology analysis. We first identified potential therapeutic targets by intersecting the targets of HL4’s active compounds with known MIRI-related genes. From a pool of 763 HL4-related targets and 1678 MIRI-related targets, we identified 422 common targets, as depicted in the Venn diagram (Figure 2A). This significant overlap suggests a strong correlation between HL4’s pharmacological actions and the pathophysiology of MIRI. To elucidate the functional relationships among these 422 common targets, we constructed a protein-protein interaction (PPI) network (Figure 2B). The network revealed a dense web of interactions, indicating that these targets function as a cohesive biological system rather than isolated entities. To pinpoint the most critical nodes within this network, we performed a topological analysis and identified the top 30 ranking hub genes. As shown in Figure 2C, key proteins such as TP53, BCL2, ESR1, PIK3R1, MAPK1, and our protein of interest, STAT3, emerged as central hubs with high connectivity, suggesting their pivotal roles in mediating the effects of HL4. To visualize the direct link between HL4’s active compounds and these key targets, we constructed a “Drug-Component-Target-Disease” network (Figure 2D). This network illustrates how multiple active components from HL4, such as Geniposide, Thiocticacid, and Genipin, are predicted to interact with a multitude of core MIRI targets, including STAT3, AKT1, JAK1/2, and PIK3CA/B. This multi-component, multi-target interaction model provides a clear visual representation of HL4’s synergistic therapeutic mechanism. Finally, to understand the biological functions governed by these core targets, we performed Gene Ontology (GO) and Kyoto Encyclopedia of Genes and Genomes (KEGG) pathway enrichment analyses. The GO analysis, encompassing Biological Process (BP), Cellular Component (CC), and Molecular Function (MF), revealed significant enrichment in processes highly relevant to MIRI (Figure 2E). Specifically, the top enriched BP terms included “cellular response to chemical stress,” “protein kinase B signaling,” and “cellular response to oxidative stress” (Figure 2F). The KEGG pathway analysis further corroborated these findings, identifying critical signaling pathways such as the “AGE-RAGE signaling pathway in diabetic complications,” “Apoptosis,” and pathways related to inflammation and viral infections, which are known to be dysregulated in MIRI. These enrichment results strongly suggest that HL4 exerts its cardioprotective effects by modulating key biological processes including oxidative stress, inflammation, and apoptosis, with the STAT3 pathway likely playing a central role in this regulatory network.

### 3.3 Systems-level analysis validates STAT3 as a central hub in an MIRI-Critical co-expression network

**FIGURE 3:**
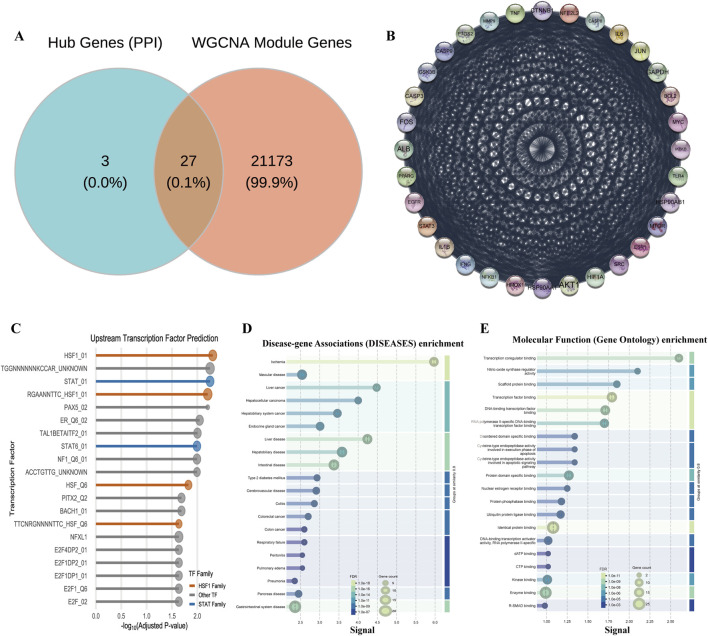
Systems biology analysis of HL4’s regulatory network in MIRI. **(A)** Venn diagram showing the intersection of hub genes and WGCNA module genes. **(B)** PPI network of the 27 core targets. **(C)** Enrichment plot of predicted upstream transcription factors for the midnightblue module. **(D)** DO enrichment analysis plot for the core targets. **(E)** GO Molecular Function enrichment analysis plot for the core targets.

To provide a systems-level validation for our previously identified targets and to systematically deconstruct the molecular network regulated by HL4, we conducted a comprehensive bioinformatics analysis of the MIRI-related dataset GSE108940. Our first objective was to identify modules of co-expressed genes that are most significantly associated with the MIRI disease state. To achieve this, we performed a Weighted Gene Co-expression Network Analysis (WGCNA) and correlated the eigengene of each of the 29 identified modules with the MIRI phenotype (see Supplementary Figure S2 for details on module generation). The resulting module-trait relationship heatmap allowed us to quantify this association. This analysis revealed that a single module, designated “midnightblue,” exhibited the strongest and most statistically significant negative correlation with MIRI status (r = −0.92, P < 0.01), pinpointing it as the most pathologically relevant gene set for our investigation. To illustrate how these modules were generated, the gene dendrogram in Supplementary Figure S2B displays the hierarchical clustering process that groups genes with similar expression patterns into distinct modules, represented by different colors. To visually confirm the collective behavior of the key midnightblue module, we generated an expression heatmap for its top 50 most altered genes (Supplementary Figure S2C). This heatmap clearly shows a coordinated pattern of downregulation for the majority of these genes in the I/R group compared to the sham group, providing direct visual evidence that supports the strong negative correlation identified in our initial analysis. Having identified the MIRI-critical midnightblue module, our next goal was to screen for the most pivotal core target genes by integrating our network pharmacology data with the WGCNA results. We employed a robust dual-network strategy for this purpose. The Venn diagram in Figure 3A visualizes this screening process, showing the intersection between our previously identified 30 hub genes (representing structural importance in the PPI network) and all genes from the WGCNA modules (representing functional co-expression). This intersection yielded a refined list of 27 final core targets. These core targets were not randomly distributed but were found to be significantly enriched in several key co-expression modules, particularly the turquoise, tan, and the MIRI-critical midnightblue module itself (Supplementary Figure S1C). To understand the functional relationships between these 27 targets, we constructed their protein-protein interaction (PPI) network (Figure 3B). The network diagram revealed that these targets form a dense and highly interconnected biological system, not merely a list of unrelated genes. Critically, within this network, our key target STAT3 was prominent as a high-degree hub, directly connecting to other pivotal molecules like AKT1 and BCL2, which strongly supports its central regulatory role in the context of MIRI.

The discovery of this STAT3-centric network led us to a crucial validation step: could we independently predict the master regulators controlling this system? We performed an upstream transcription factor (TF) prediction analysis on the genes within the MIRI-critical midnightblue module. The resulting lollipop plot (Figure 3C) displays the most significantly enriched TFs predicted to regulate this module. This analysis yielded a striking result: the STAT family of transcription factors (e.g., STAT_01, Adjusted P-value = 0.0056) was significantly enriched. This independent bioinformatic prediction, which identifies STAT TFs as “master switches” for this MIRI-related gene set, provides powerful corroboration for our experimental findings and reinforces the centrality of the STAT3 pathway. Finally, to anchor our findings in a clear biological and clinical context, we conducted comprehensive functional enrichment analyses on the 27 core targets. To assess their clinical relevance, we performed a Disease Ontology (DO) enrichment analysis. The results, shown in Figure 3D, were compelling: “Ischemia” emerged as one of the most significantly enriched disease terms (FDR = 7.16e-16), directly validating that our core targets are fundamentally linked to the disease pathology. To understand their biological roles, we then performed a Gene Ontology (GO) analysis for molecular function. As shown in Figure 3E, this revealed an overwhelming enrichment in functions related to transcriptional control, such as “Transcription factor binding” (FDR = 1.12e-09). This finding aligns perfectly with our upstream TF prediction, collectively indicating that these core targets primarily function by modulating gene expression at the transcriptional level.

In summary, our multi-step systems biology analysis provides a powerful, independent validation of our experimental results. It began by identifying a MIRI-critical gene module, then screened for a core network of 27 targets with STAT3 at its center, and culminated in the crucial bioinformatic discovery that the STAT family is a key upstream regulator of this network. These findings collectively establish a strong evidence-based foundation for our central hypothesis: HL4 exerts its cardioprotective effects through the modulation of a STAT3-centric transcriptional regulatory network.

### 3.4 Molecular docking validates strong binding affinity between key HL4 compounds and STAT3

**FIGURE 4:**
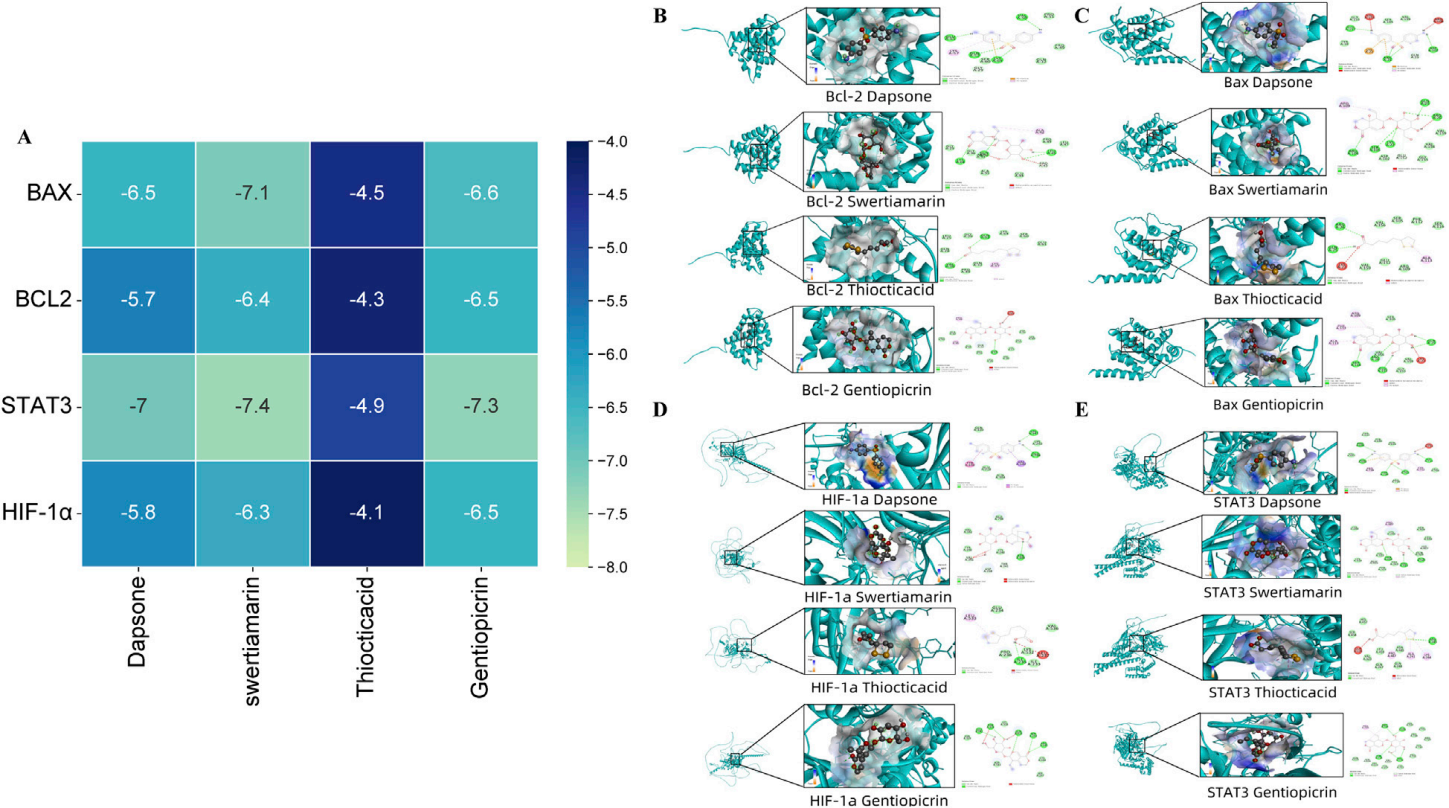
Molecular docking of HL4 compounds with key target proteins. **(A)** Heatmap of docking scores (kcal/mol) for four compounds against four target proteins. **(B)** Binding poses of compounds with BCL2. **(C)** Binding poses of compounds with BAX. **(D)** Binding poses of compounds with HIF-1α. **(E)** Binding poses of compounds with STAT3. Each sub-panel in **(B–E)** displays the 3D docking conformation and the corresponding 2D diagram of intermolecular interactions.

To further investigate the interaction between the active compounds of HL4 and our experimentally validated core targets at an atomic level, we performed molecular docking simulations. We selected four representative active compounds (Dapsone, swertiamarin, Thiocticacid, and Gentiopicrin) and docked them with four key target proteins (BAX, BCL2, STAT3, and HIF-1α). The binding affinities, expressed as docking scores (kcal/mol), are summarized in the heatmap in Figure 4A. The docking scores for all 16 compound-target pairs ranged from −4.1 to −7.4 kcal/mol, with lower scores indicating stronger binding affinity. Our analysis revealed that all four compounds exhibited favorable binding potential with the target proteins. Notably, the interaction between swertiamarin and STAT3 yielded the lowest docking score of −7.4 kcal/mol, suggesting the strongest and most stable binding among all tested pairs. Focusing on our primary target, STAT3, it demonstrated strong interactions with multiple compounds, exhibiting binding energies of −7.0 kcal/mol with Dapsone, −7.4 kcal/mol with swertiamarin, and −7.3 kcal/mol with Gentiopicrin. In contrast, the binding affinities with Thiocticacid were generally weaker across all targets, with the score for the STAT3-Thiocticacid pair being −4.9 kcal/mol. These results from the binding energy calculations strongly suggest that compounds like swertiamarin have a high potential to be key active molecules that directly engage STAT3. To visualize the precise interaction modes, we examined the 2D and 3D docking poses. As a representative example of a high-affinity interaction, the docking model of swertiamarin with STAT3 is shown in Figure 4E. The 3D view shows swertiamarin deeply embedded within the binding pocket of the STAT3 protein. The corresponding 2D interaction diagram reveals that this stable binding is maintained by a network of specific intermolecular forces. For instance, swertiamarin forms multiple conventional hydrogen bonds with the amino acid residues GLN 635, SER 611, and ASN 647 of STAT3. Additionally, the complex is further stabilized by several carbon-hydrogen bonds and van der Waals forces with surrounding residues. Similar strong interactions were observed for other key pairs, such as Dapsone with STAT3 (Figure 4E) and swertiamarin with BAX (Figure 4C, score −7.1 kcal/mol).

In summary, the molecular docking results provide compelling computational evidence that corroborates our experimental findings. The strong and specific binding interactions observed, particularly between key active compounds and STAT3, strongly support the hypothesis that HL4 exerts its therapeutic effects in MIRI through the direct modulation of the STAT3 signaling pathway.

### 3.5 HL4 protects H9c2 cardiomyocytes and modulates the STAT3 signaling pathway in an *in vitro* model of myocardial ischemia-reperfusion injury

**FIGURE 5:**
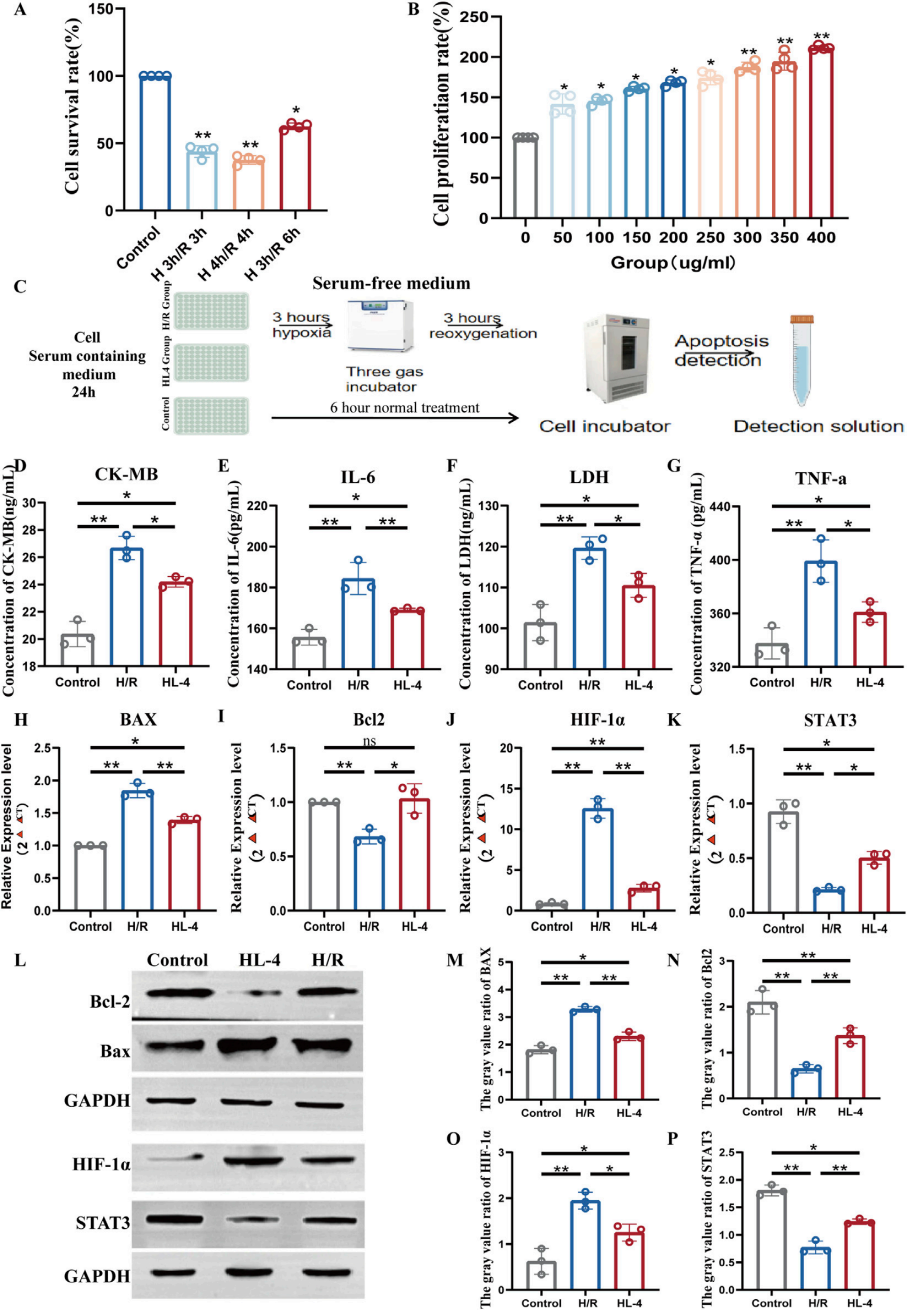
HL4 protects H9c2 cardiomyocytes from hypoxia/reoxygenation (H/R) injury by activating the STAT3 signaling pathway. **(A)** Cell viability of H9c2 cardiomyocytes under different H/R time courses, used to establish the injury model. **(B)** Dose-dependent effect of HL4 on H9c2 cardiomyocytes proliferation, determined by CCK-8 assay to identify the optimal concentration. **(C)** Schematic diagram illustrating the experimental workflow, including the serum-free hypoxia step. **(D–G)** HL4 pre-treatment significantly reduced the H/R-induced release of the injury markers **(D)** CK-MB and **(F)** LDH, and the pro-inflammatory cytokines **(E)** IL-6 and **(G)** TNF-α. **(H–K)** qRT-PCR analysis showing that HL4 reversed the H/R-induced changes in the mRNA expression of **(H)** Bax, **(I)** Bcl-2, **(J)** HIF-1α, and **(K)** STAT3. **(L)** Representative Western blot images of key proteins in the control, H/R, and HL4-treated groups. GAPDH served as the loading control. **(M–P)** Densitometric quantification of protein levels for **(M)** Bax, **(N)** Bcl-2, **(O)** HIF-1α, and **(P)** STAT3, confirming the trends observed at the mRNA level. All data are presented as mean ± SEM from at least three independent experiments. *P < 0.05, **P < 0.01.

To investigate the cardioprotective effects of HL4 *in vitro*, we first established a robust hypoxia/reoxygenation (H/R) injury model. We found that subjecting H9c2 cardiomyocytes to 3 h of hypoxia followed by 3 h of reoxygenation (3h H/R) significantly reduced cell viability to 45.8% ± 4.2% compared to the normoxic control group (Figure 5A, P < 0.01), confirming the establishment of a significant and reproducible injury model. We then determined the optimal therapeutic concentration of HL4 via a CCK-8 assay. As shown in Figure 5B, HL4 treatment (0–400 μg/mL) elicited a clear dose-dependent increase in cell proliferation. For instance, cell proliferation increased from 145% at 50 μg/mL to 180% at 150 μg/mL. Although proliferation continued to rise modestly at higher concentrations, reaching approximately 210% at 400 μg/mL, the rate of increase markedly slowed after 150 μg/mL, indicating that the dose-response effect was entering a plateau phase. Given that 150 μg/mL already conferred a robust and statistically significant protective effect, we selected this concentration as the optimal balance between efficacy and dosage for all subsequent experiments (workflow depicted in Figure 5C). Pre-treatment with 150 μg/mL HL4 conferred potent protection against H/R-induced cellular damage and inflammation. In the H/R model group, the concentration of the cardiac injury marker creatine kinase-MB (CK-MB) surged to 26.5 ng/mL, a significant increase from the control level of 20.3 ng/mL (Figure 5D, P < 0.01). HL4 pre-treatment effectively reduced this level to 24.1 ng/mL. Similarly, the level of lactate dehydrogenase (LDH), an indicator of cell membrane damage, rose from 101 ng/mL to 120 ng/mL after H/R, an effect that was significantly attenuated by HL4 (Figure 5F, P < 0.01). Concurrently, H/R induced a strong inflammatory response, elevating IL-6 levels from 150 pg/mL to 210 pg/mL (Figure 5E, P < 0.01) and TNF-α levels from 330 pg/mL to 400 pg/mL (Figure 5G, P < 0.01). HL4 pre-treatment markedly suppressed the secretion of both IL-6 and TNF-α, demonstrating its strong anti-inflammatory properties.

To elucidate the underlying molecular mechanisms, we first examined gene expression at the transcriptional level. Our primary finding centered on the pro-survival factor STAT3, whose relative mRNA expression was drastically reduced by over 75% in the H/R group compared to controls (Figure 5K, P < 0.01). Strikingly, HL4 pre-treatment caused a nearly three-fold increase in STAT3 mRNA compared to the H/R group, significantly rescuing its expression. Consistent with this restoration, the expression of the anti-apoptotic gene Bcl-2, which was halved by H/R, was fully restored by HL4 (Figure 5I, P < 0.01). Conversely, the expression of the pro-apoptotic gene Bax nearly doubled in the H/R group, an effect that was almost completely reversed by HL4 (Figure 5H, P < 0.01). Furthermore, the maladaptive hypoxia response factor HIF-1α, whose expression was induced by more than 12-fold during H/R, was suppressed by over 60% following HL4 treatment (Figure 5J, P < 0.01).

To confirm these findings at the protein level, we performed Western blot analysis (Figure 5L). The densitometric quantification corroborated the transcriptional data, showing that H/R stress led to a profound decrease in STAT3 protein expression by approximately 60%, which was significantly restored by HL4 (Figure 5P, P < 0.01). This reactivation of STAT3 was accompanied by the expected downstream effects: the protein level of anti-apoptotic Bcl-2 was significantly increased by HL4 (Figure 5N, P < 0.01), while the level of pro-apoptotic Bax was markedly decreased (Figure 5M, P < 0.01). Finally, consistent with the mRNA data, the H/R-induced surge in HIF-1α protein was also significantly suppressed by HL4 treatment (Figure 5O, P < 0.01).

Taken together, these comprehensive quantitative data demonstrate that HL4 alleviates myocardial H/R injury by activating the STAT3 signaling pathway, which in turn orchestrates a downstream anti-apoptotic program by robustly increasing the Bcl-2/Bax ratio and inhibits the deleterious HIF-1α response.

## 4 Discussion

This study sought to elucidate the precise molecular mechanisms by which the traditional Mongolian formula HuangLian-4 (HL4) protects against myocardial ischemia-reperfusion injury (MIRI). Our principal finding is that HL4 mitigates hypoxia/reoxygenation-induced cardiomyocyte apoptosis by activating the pro-survival STAT3 signaling pathway. This activation, in turn, orchestrates a downstream protective program characterized by the suppression of the pro-apoptotic protein Bax and the maladaptive factor HIF-1α, alongside an upregulation of the anti-apoptotic protein Bcl-2. While previous studies have attributed the cardioprotective effects of other herbal interventions, or individual components like berberine, to pathways such as PI3K/Akt or Nrf2, our results identify the STAT3/HIF-1α axis as a distinct and central mechanism for the therapeutic action of the complete HL4 formula (Chen et al., 2014; Dong et al., 2023; Ferdinandy et al., 2023). This distinction is critical; it suggests that while individual constituents may act on certain pathways, the synergistic effect of the entire formula converges on STAT3 signaling to exert its primary protective effect in this MIRI model, thereby adding a new layer of understanding to the pharmacology of this traditional remedy (Chunhua et al., 2017; Fabritius and Yang, 2023).

UPLC-QTOF-MS analysis identified 40 blood-absorbed components from HL4, including alkaloids, terpenoids, and phenylpropanoids. Alkaloids such as berberine are renowned for their potent antioxidant and anti-inflammatory effects, reducing cardiomyocyte apoptosis by attenuating oxidative stress and inflammation in ischemia-reperfusion injury (Heusch, 2020; Krittanawong et al., 2023). Terpenoids and phenylpropanoids often function as signaling modulators, influencing cell survival and apoptosis pathways (Gauthier et al., 2019; Luo et al., 2023). The diverse chemical profile underpins HL4’s complex pharmacology, supporting the hypothesis of multi-component synergy *in vivo*.

Network pharmacology revealed 422 shared targets between HL4 and MIRI, with a PPI network highlighting hub genes including TP53, a pivotal tumor suppressor regulating cell cycle and apoptosis, crucial for cardiomyocyte survival under stress (Zhao et al., 2023; Tang et al., 2025). Anti-apoptotic BCL2 and pro-apoptotic Bax form a key rheostat controlling cell fate during MIRI (Tavakoli et al., 2021; Xiang et al., 2024). ESR1 mediates cardioprotection via antioxidant gene regulation and inflammation suppression (Xie et al., 2021). PIK3R1 and MAPK1 are central to the PI3K/Akt and MAPK pathways governing proliferation, survival, and stress response. STAT3 is a master regulator promoting anti-apoptotic gene expression and limiting inflammation (Yamashita et al., 2024). The enrichment of these targets indicates HL4’s multi-pathway modulation of complex myocardial injury processes.

WGCNA identified the “midnightblue” module strongly negatively correlated with MIRI (r = −0.92, P < 0.01), with significant downregulation in I/R conditions indicating loss of protective gene expression. STAT3 sits at the network center, closely connected with AKT1 and BCL2. AKT1, a key kinase in the PI3K/Akt pathway, promotes survival and inhibits apoptosis, acting synergistically with STAT3 to reduce ischemia-induced cardiomyocyte death (Zhang et al., 2024). Transcription factor enrichment highlights STAT family members as principal regulators of this module. Disease ontology and GO analyses confirm clinical relevance and transcriptional regulation roles, reinforcing the integrative mechanism whereby HL4 activates STAT3 to orchestrate anti-apoptotic and anti-inflammatory gene networks.

Molecular docking demonstrates that active compounds such as swertiamarin bind STAT3 with high affinity, stabilized by multiple hydrogen bonds and van der Waals forces, potentially modulating STAT3 conformation and transcriptional activity through interaction with its SH2 domain (Zhao et al., 2023). Bax and Bcl-2 also show favorable binding energies, supporting HL4’s multi-target synergy in regulating cell fate. HIF-1α, a hypoxia-inducible factor critical in metabolic adaptation and pathological remodeling during ischemia, is known to be regulated by STAT3; its inhibition mitigates ischemic injury (Zheng et al., 2021). These molecular insights support a multi-faceted mechanism, suggesting that active compounds in HL4 directly bind to and modulate STAT3 activity, which in turn functions as a transcription factor to orchestrate the observed changes in downstream gene expression.

In H9c2 cardiomyocytes, HL4 markedly enhanced cell viability under hypoxia/reoxygenation stress, reduced release of injury markers CK-MB and LDH, preserving membrane integrity and function. The suppression of inflammatory cytokines IL-6 and TNF-α indicates attenuation of the inflammatory cascade typical of ischemia-reperfusion (Wang et al., 2009). Furthermore, the central role of the NLRP3 inflammasome in MIRI is well-established (Toldo et al., 2022; Toldo and Abbate, 2024). Given that STAT3 activation can negatively regulate the NLRP3 inflammasome, it is plausible that the observed anti-inflammatory effects are also mediated through this axis, which warrants further investigation. Upregulation of STAT3 and Bcl-2, with concomitant downregulation of Bax and HIF-1α, demonstrates HL4’s activation of STAT3 signaling promoting survival and inhibiting apoptosis and inflammation. These findings are congruent with network pharmacology and docking predictions, underscoring HL4’s systemic modulation of cardiomyocyte fate through a multi-component, multi-target mechanism. A key aspect of STAT3 function is its activation via phosphorylation. While our study provides strong indirect evidence for STAT3 activation by demonstrating the modulation of its canonical downstream target, Bcl-2, we acknowledge that a direct measurement of phosphorylated STAT3 (p-STAT3) was not performed. This represents a limitation of the current study. The observed rescue of total STAT3 expression by HL4, however, is a critical prerequisite for its subsequent activation. Future studies focusing on the phosphorylation status of STAT3 will be essential to definitively confirm this activation mechanism. As highlighted by important research in the field (Comita et al., 2021), the activation of the STAT3 signaling pathway is a central node in cardioprotection, and our findings strongly suggest that HL4 engages this critical pro-survival axis.

Despite these advances, limitations remain. The *in vitro* H9c2 cardiomyocytes model cannot fully replicate the complex *in vivo* microenvironment involving intercellular crosstalk and pharmacokinetics; thus, preclinical animal validation is necessary. In addition, our UPLC-QTOF-MS analysis was primarily qualitative and did not determine the absolute plasma concentrations of the identified components. The *in vitro* concentration used (150 μg/mL) was based on a dose-response curve to achieve an optimal biological effect, a common approach for complex extracts, though it may not directly reflect the *in vivo* physiological concentrations of individual compounds. The specific active compound(s) responsible for STAT3 activation within the HL4 mixture remain unidentified. Validating the effects of individual key compounds predicted by our analysis, such as swertiamarin, would be a critical next step, warranting bioactivity-guided fractionation and functional screening. Furthermore, the mechanisms by which HL4 components modulate upstream kinases of STAT3, such as JAK family members, require further elucidation. Finally, our study focused on a pre-treatment regimen to investigate protective mechanisms. Future work should incorporate a post-treatment (therapeutic) model to better assess the clinical translational potential of HL4. In summary, this study systematically elucidates HL4’s cardioprotective mechanism via the STAT3/HIF-1α axis, providing a robust scientific foundation for its clinical application and laying groundwork for developing STAT3-targeted therapies for myocardial ischemia-reperfusion injury.

## 5 Conclusion

This research demonstrates that the traditional Mongolian formula HL4 effectively mitigates myocardial ischemia/reperfusion injury by activating the pivotal pro-survival signaling pathway mediated by STAT3. The HL4-induced activation of STAT3 orchestrates a robust anti-apoptotic program, evidenced by an increased Bcl-2/Bax ratio, and concurrently suppresses the detrimental HIF-1α response in cardiomyocytes. Collectively, these findings provide a strong scientific rationale for the clinical application of HL4 and establish STAT3 modulation as a promising therapeutic strategy for the treatment of myocardial ischemia-reperfusion injury.

## Data Availability

The raw data presented in the study are deposited in figshare and the details are as follows: https://figshare.com/s/2d32394805d2b8e60c53.
