# Supplementary material for: HuangLian-4 alleviates myocardial ischemia-reperfusion injury by activating the pro-survival STAT3 signaling pathway
Source: Front Pharmacol. 2025 Nov 5;16:1683575. doi: 10.3389/fphar.2025.1683575 (PMC12626919; doi:10.3389/fphar.2025.1683575)
Supplement: Supplementary file 1 [file Supplementaryfile1.doc]

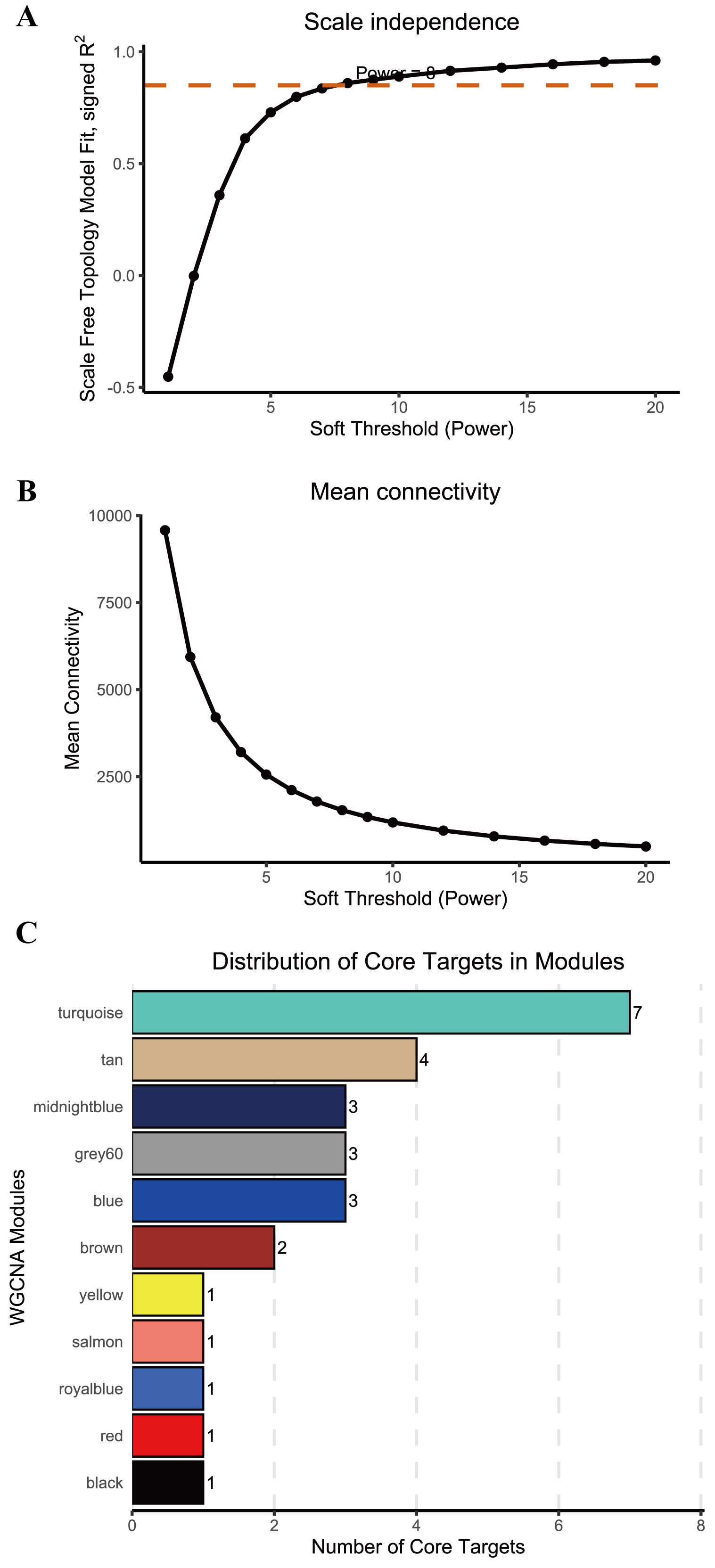


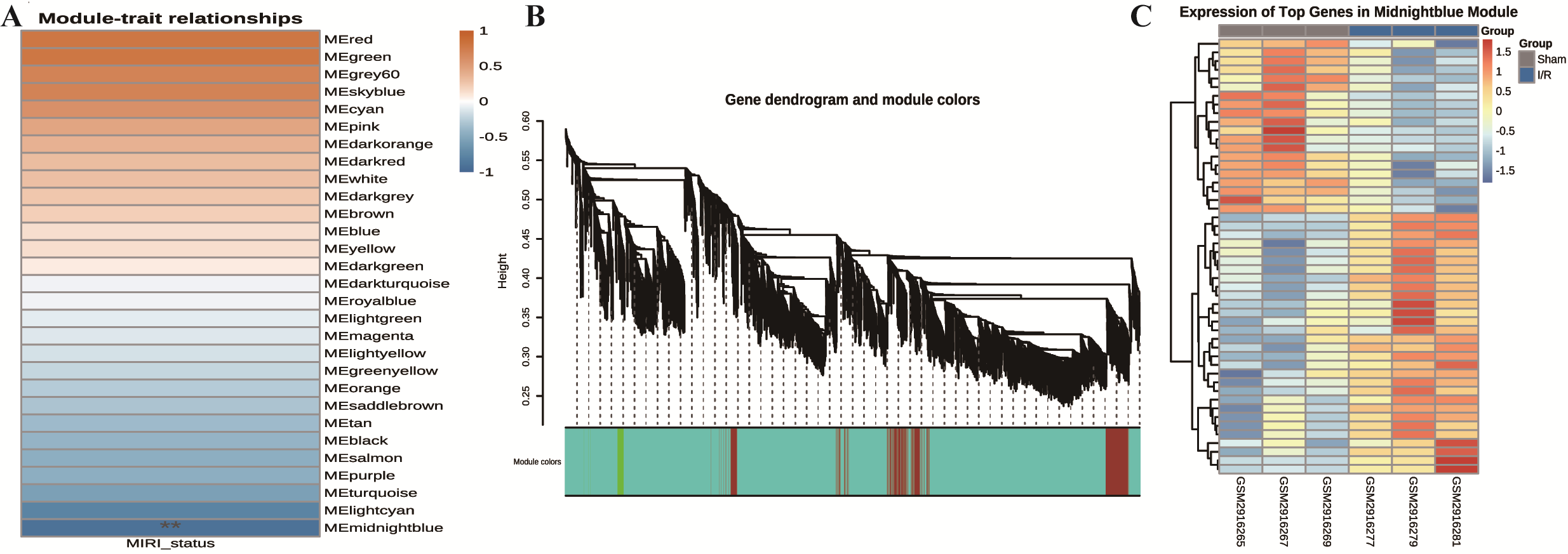
Figure S1. Parameter selection for WGCNA and distribution of core targets.
(A) Analysis of the scale-free fit index for various soft-thresholding powers (β). The red dashed line indicates the recommended R² threshold of 0.85. A power of β = 8 was chosen.(B) Analysis of the mean connectivity for various soft-thresholding powers.
(C) Bar plot showing the distribution of the 27 core targets across the WGCNA co-expression modules.

Figure S2. (A) Heatmap of module-trait relationships from WGCNA.(B) Gene dendrogram and corresponding module color assignments.(C) Expression heatmap of the top 50 genes in the midnightblue module.
